# The Title of "Physician"

**Published:** 1899-02-04

**Authors:** 


### The Hospital

A Journal of

The Medical Sciences and Hospital Administration.

Vol. XXV.—No. 645.] Edited by Sir Henry Burdett, K.C.B., and Solomon C. Smith, M.D., M.R.C.P. [Feb. 4, 1899.

### The Title of "Physician."

The accumulating evidence in favour of amendment of the Medical Acts was greatly strengthened last week by the *obiter dicta* of Mr. Justice Lawrance and Mr. Justice Channell on the question whether a licentiate in medicine and surgery of the Society of Apothecaries was entitled to describe himself as a "physician." The question arose in the case of a gentleman who possessed the licence of the Society, together with an American doctorate which, at the time, was not registrable in England. He was complained of by a neighbouring practitioner for calling himself "M D," but before the necessary summons was applied for the defendant discontinued the practice complained of, and the local justices refused to grant a summons with reference to the abandoned "M.D.," but offered to do so with reference to the use of the word "physician." This offer was accepted, and the magistrates convicted the defendant, for describing himself as a "physician," of an offence against the 40th Section of the Medical Act of 1858. The conviction was appealed against, and, although the defendant had in the meantime died, the appeal was prosecuted for the purpose of determining the law of the case. It was heard on the 24th ult., in the Queen's Bench Division, and ended in determining nothing. The judges quashed the conviction on the ground that what the defendant had done had not been done "wilfully and falsely;" and although they expressed opinions as to the right of licentiates of the Society to describe themselves as "physicians," these opinions formed no part of the judicial decision in the case, which turned solely upon the "wilfully and falsely," and they were, therefore, as mentioned above, only *obiter dicta*, of no legal authority. Mr. Justice Lawrance was of opinion that a Licentiate "was not entitled so to describe himself," and Mr. Justice Channell did not think there was "any binding authority as to the meaning of physician in the section," and that everything depended on the sense in which the word was used.

It appears to us, as a matter of common sense. that a "physician" must be one of two things, He must either be an actual member of a corporation of physicians, in which case the mere "licentiates" of such corporations, not being "members," can have no claim to the designation, or else he must fulfil the terms of the definition given by Johnson, and be "one who professes the art of healing." The various Colleges of Physicians in the United Kingdom derive various powers from ancient Charters, powers the extent and limitations of which are probably known only to their respective lawyers; but it may fairly be doubted whether they are entitled to appropriate to themselves, and, as it were, to monopolise, an English word of greater antiquity than these Charters, or to warn off all other persons from the use of it. The intention of the Legislature, in casting upon the Medical Council the duty of securing that all qualifying examinations for the medical profession shall be maintained at an equal standard of efficiency, was clearly that all such examinations should confer equal privileges. Before the Act of 1858, as Mr. Justice Channell truly observed, the word "physician" was commonly used as applying to persons in the highest grade of medical practitioners; and it is quite intelligible that the colleges of physicians should cling to the idea that they can render their merely qualifying examination more popular by attaching to it a title which implies, although at the present time erroneously, the attainment of this "highest grade." It does not seem to be in the public interest that they should do so; nor is it in the public interest that the Licentiates of the Society of Apothecaries, who pass through precisely the same curriculum of study as the Licentiates of the College of Physicians, and who, unless the Medical Council fails in the discharge of its most important duty, undergo the test of a precisely equivalent examination, should only be allowed to assume a designation which, in the minds of many people, is associated with a professional grade inferior to that of the physician. The word "apothecary" has long ceased to have any meaning in this country, and, moreover, it may fairly be doubted whether any but yeomen or liverymen of the Society are entitled to use it. The words "Licentiate in

Medicine and Surgery of the Society of Apothecaries" are too long and cumbrous for ordinary purposes; and a designation is required which shall be the common property of every legally qualified medical practitioner, without regard to the body from which his qualifications were obtained. The simple words "physician" and "surgeon" seem to meet this requirement better than any others which can be devised; and it is to be hoped that the right to use them will soon be universally recognised.

